# Factors associated with the public’s trust in physicians in the context of the Lebanese healthcare system: a qualitative study

**DOI:** 10.1186/s12913-019-4354-0

**Published:** 2019-07-27

**Authors:** Bashir Shaya, Nadine Al Homsi, Kevin Eid, Zeinab Haidar, Ali Khalil, Kelly Merheb, Gladys Honein-Abou Haidar, Elie A. Akl

**Affiliations:** 1 School of Medicine, American University of Beirut, P.O. Box: 11-0236, Riad-El-Solh Beirut, Beirut, 1107 2020 Lebanon; 2 Hariri School of Nursing, American University of Beirut, P.O. Box: 11-0236, Riad-El-Solh Beirut, Beirut, 1107 2020 Lebanon; 3 Department on Internal Medicine, American University of Beirut Medical Center, P.O. Box: 11-0236, Riad-El-Solh Beirut, Beirut, 1107 2020 Lebanon

**Keywords:** Trust, Physician-patient relationship, Lebanon

## Abstract

**Background:**

The Lebanese public perceives the physician-patient relationship as flawed. The objectives of this study are to assess factors associated with the public’s trust in physicians in the context of the Lebanese healthcare system and to explore potential ways to enhance it.

**Methods:**

We conducted a qualitative study based on a grounded theory methodology using semi-structured interviews with members of the Lebanese public (not restricted to patients). We selected participants through convenience and maximum variation sampling approaches. The constant comparative analysis resulted in a theoretical framework that describes the factors influencing trust in physicians.

**Results:**

Participants trusted an experienced, up-to-date, graduate of a North American or Western European school, working in a reputable hospital, with a high level of diagnostic skills. The personal characteristics that improved trust were physicians who are ‘non-materialistic’, have a good rapport, and have sufficient encounter time with patients. Social factors that enhance trust in the physician include: being a family member, recommended by a family member, featured in mainstream media, and/or having a good reputation. Trust increased compliance, loyalty despite occasional mistakes committed, high consultation fees, and negative attitudes towards the physician’s institution. Conversely, no trust led to severed therapeutic relationship and seeking second opinions.

**Conclusion:**

The level of trust of members of the Lebanese public in physicians was affected by the personal characteristics of physicians, their practice or clinical skills, their interactions with the patient, finances, in addition to a number of social factors. Moreover, the level of trust had major implications on patients’ interactions with their physicians.

**Electronic supplementary material:**

The online version of this article (10.1186/s12913-019-4354-0) contains supplementary material, which is available to authorized users.

## Background

Trust can be defined “as a state of favourable expectation regarding other people’s actions and intentions” [1], Simmel argues that trust concerns “a state of mind which has nothing to do with knowledge, which is both less and more than knowledge” [2]. While Luhmann suggests that “trust occurs in a framework of interaction which is influenced by both personality and social systems and cannot be exclusively associated with either” [3] similar to Giddens who argues that trust in individuals is dependent on “trust in a variety of social systems” [3].

Trust is an essential building block in the patient-physician relationship. Pearson and colleagues describe trust in physicians as “a reassuring feeling of confidence or reliance in the physician and the physician’s intent” [4]. Moreover, Crocker and colleagues propose that to trust physicians, patients expect them to be proficient, well-informed, experienced, skilful; to hold the patient’s best interest above everything else; and to get the sense of ‘being taken seriously’ [5].

Trust does not arise from one encounter and is not “blind faith” [6]. It develops gradually with repeated interactions where the patient explores all necessary means to trust their doctor. Hall and colleagues compare the way trust defines the patient-physician relationship to “the way love or friendship defines the quality of an intimate relationship” [7]. They propose a five-part configuration of trust: fidelity, honesty, competence, confidentiality, and global trust [7]. In a more recent study, Hillen and colleagues discuss similar correlates of trust which overlap with the aforementioned configuration [8].

Patients’ trust in a physician contributes to a long-standing relationship with that same physician for their services and care. In such cases, the physician knows the patient’s medical history, can identify their risk factors more accurately, and can closely monitor their health status. From the patient’s perspective, trust in their physicians increases the patient compliance to treatment [7]. Even with the patient’s limited knowledge on the best course of treatment, they find themselves willing to yield to their physician’s choice of action. Moreover, when patients are in a vulnerable state due to illness, uncertainties and fear, they tend to become completely dependent on the physician [9]. This is corroborated by Hall and colleagues who argue that trust is further accentuated in situations when the vulnerability of the patient increases [7].

Globally, trust in physicians is in decline [10], which threatens the physician-patient relationship and eventually subjects the health care system to dire consequences. For example, Armstrong and colleagues report that distrust in the health care system in the United States is common among its population [11]. The “Golden age of doctoring” has lasted from the twentieth century to the 1970s. In the following decades, American trust in the system declined significantly [12]. Similarly, a study conducted in China tracks the decline in trust of physicians among patients [13].

In Lebanon, public trust in physicians is also in decline. Arawi and colleagues indicate that the Lebanese public perceives the physician-patient relationship as flawed, “characterized by materialistic gain and a feeling of being treated as a ‘number’ and not as a ‘person’” [14]. However, the study did not explore the reasons underlying these negative perceptions, nor did it explore ways to improve those perceptions.

Therefore, the objectives of this study are to assess the factors associated with members of the Lebanese public’s trust in physicians in the context of the Lebanese healthcare system and to explore potential ways to enhance it.

## Methods

### Study overview

In this study we opted for an exploratory qualitative design based on Corbin and Strauss’ grounded theory approach to provide insights on the social processes that were associated with the participants’ trust in physicians [15]. Consistent with this method, our sampling, data collection, and data analysis occurred simultaneously [15]. A semi-structured interview guide was used in in-depth interviews during data collection and the constant comparative approach was used during data analysis [15].

We followed the Consolidated Criteria for Reporting Qualitative Research (COREQ) in drafting this manuscript [16]. COREQ guidelines include a 32-item checklist for reporting important aspects of the research team, study methods, context of the study, findings, analysis and interpretations. The Ethical Review Committee at the American University of Beirut approved this study.

### The research team

Two team members (GHA and EAA), with significant experience in conducting and publishing qualitative studies, overlooked the research. The rest of the team (NA, KE, ZH, AK, KM, BS) were second-year medical students with no previous experience in qualitative research when the study was conducted. They were trained in interviewing and data analysis techniques. Following the first few interviews, debriefing sessions were held to reflect on the process of data collection and measures for improvement.

### Population, sampling strategy and recruitment

Our target population consisted of adult (ages 18 and above) Lebanese nationals residing in Lebanon. We did not restrict the population to ‘patients’ as we were seeking perspectives related to the wider general population. We included Arabic and English speakers from this population as the interview and consent form were only available in these two languages. We recruited participants from different governorates, residential areas (urban, suburban and rural), and demographic profiles (i.e., age, sex, and level of education). We also sought participants from public places (e.g., minimarkets, supermarkets, malls, coffee shops) and eligible individuals living in the co-investigators’ neighbourhoods to participate in our study.

Our sampling was guided by the intention of approaching participants who would count as informant-rich sources. Therefore, we identified and approached eligible members of the Lebanese public through both convenience and maximum variation sampling approaches. To increase heterogeneity, we captured participants that varied by governorate, gender, and age. We made sure that the interviewer and interviewee had no prior relationship. Individuals interested in the study were asked for oral consent and were given at the time of the interview an information sheet that did not require a signature. Abiding by the grounded theory approach, sampling was concurrent with data collection and analysis. We proceeded with recruitment until no further themes emerged (thematic saturation) [15].

### Data collection

We conducted face-to-face in-depth interviews throughout the months of February and March 2017. We chose settings convenient to participants for conducting the interviews. We audiotaped each interview; when participant refused to consent, we resorted to note-taking. We used the interviewee’s language of preference (Arabic or English). Each interview lasted between 15 and 30 min.

The interview started with a short questionnaire on the participant’s demographic characteristics (See Additional file 1). We used a semi-structured interview to guide the discussion (See Additional file 2), starting with a grand tour question reflecting on their perception of trust in physicians in context of the Lebanese healthcare system followed by more specific questions focusing on the different factors associated with that trust, as well as potential ways to enhance it. However, we often departed from the interview guide to focus on the interviewee’s prompts and opinions, hence allowing new ideas to emerge.

### Data management

We assigned a unique ID code for each participant using the following format: P01, P02, P03, etc. On a separate log, we recorded each participant’s demographic characteristics and the date of the interview. This log sheet was kept in a secured double password file on the Principal Investigator’s (PI) computer. We transcribed audio-recorded interviews conducted in English. Arabic interviews were translated as we were working on the transcription. We proceeded with the processes of transcription, translation, and data analysis immediately following each interview.

### Data analysis

Consistent with an inductive constant comparative approach, the three pairs of investigators conducting the interviews independently read, categorized, labelled, and coded line by line the first three transcripts (open coding). All the aforementioned co-investigators met with the two senior investigators to share, analyse, compare, and contrast their results (constant comparative technique). We repeated the same process for the second and third set of three interviews and then compared their results with the results of the three previous interviews. This comparison allowed us to identify commonalities and variations when we started eliciting categories from the data.

We used these categories to refine the interview guide which was used in the following interviews, as it gave way to more probing and clarified issues which were, otherwise, still uncovered. These subsequent interviews were coded line by line, and their results were compared with each other. The categorization following this process allowed the elicitation of the emerging themes (axial coding). We continued the data collection until we reached data saturation (i.e., no more new themes emerged). Then, we stopped the data collection and met to discuss our final findings and to develop the theoretical framework weaving the thematic categories into an integrated whole. The integrated framework shows the factors associated with the trust of members of the public in physicians in the context of the Lebanese healthcare system (selective coding) [15]. Significantly, throughout the analysis process, we discovered that the physician’s country of training is tied to the patients’ trust in this physician (open coding). Thus, by comparing all the interviews, we found that patients have more trust in physicians trained in Western Europe or North America as opposed to Eastern Europe.

### Increasing rigour

We worked hard to refine our findings’ credibility (i.e. referring to confidence in the truth of the data and interpretations of them) and confirmability (i.e. when the data represents the information which the participants provided and that the interpretations of this data are not based on the inquirer’s bias, motivation, or interest) [17]. In terms of credibility, we used transcribed audio-recorded interviews as the main data repository, and we made sure we shared with the participants our experience, credentials and motivation (researcher credibility). During data analysis, we maintained an audit trail in the form of a log of emerging categories following each constant comparative analysis. For data reporting, we supported the narrative by quotes from participants. In terms of confirmability, we had two independent researchers coding the transcripts. Then, we all examined the congruence between them in terms of meaning. Team members maintained a high level of objective reflexivity to prevent bias during data collection, data analysis, and research writing stages. We also worked as a team in the analysis to avoid bias in the interpretations of the results.

## Results

All individuals approached for the study agreed to participate. We recruited a total of 27 individuals from 4 of the 6 governorates in Lebanon. Table 1 provides a summary of the demographic characteristics of those participants. All the participants had a minimum of elementary school education, and 52% reached the university level. Among the participants, 63% reported seeing a physician in the past 4 weeks.

**Table 1:** General demographic characteristics of the participants

Variable	N (%)
Gender	
Male	13 (48%)
Female	14 (52%)
Age	
18–30	5 (19%)
31–40	6 (22%)
41–50	4 (15%)
51–60	9 (33%)
> 60	3 (11%)
Governorate	
Beirut	5 (19%)
Bekaa	11 (41%)
Mount Lebanon	9 (33%)
South	2 (7%)
Educational status	
Elementary	5 (19%)
High school	8 (30%)
Undergraduate	12 (44%)
Masters/PhD	2 (7%)
Physician visit within past 4 weeks	
Yes	17 (63%)
No	10 (27%)

**Fig. 1:**
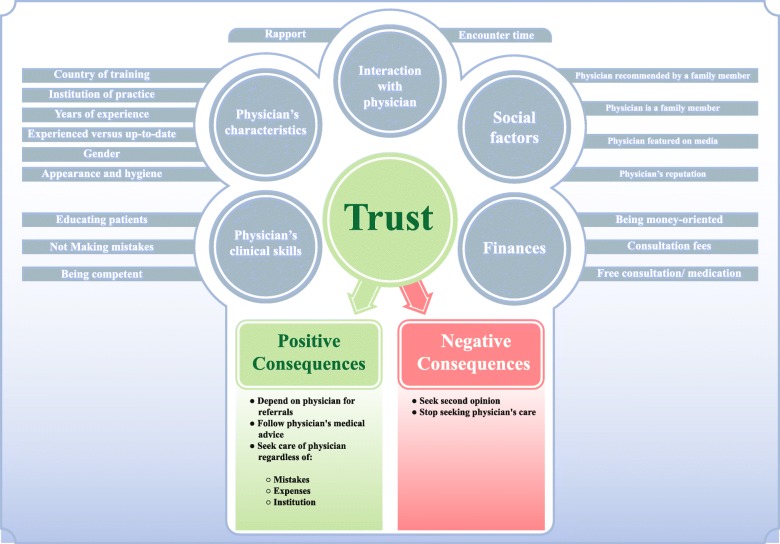
Entitled “Graphical representation of factors perceived as associated with the trust of members of the public in physicians in the context of the Lebanese healthcare system, and the consequences of increased and decreased trust” is the theoretical framework that summarizes the different factors associated with trust and how it interact based on our results

Our analysis identified factors associated with individuals’ trust in physicians in the context of the Lebanese healthcare system. Additional file 3 contains all the themes, subthemes and codes. Table 2 lists these factors and indicates whether each factor increases, has no effect on, or decreases trust in physicians. We categorized these factors under five main themes: physician’s personal and practice characteristics, physician’s clinical skills, interactions with the physician, finances, and social factors. Figure 1 depicts the theoretical framework that describes those factors and how they relate. We report on these factors as well as the related implications in detail in the next sections.

**Table 2:** Factors associated with the trust of members of the public in physicians in the context of the Lebanese healthcare system

Factors	Perceived impact on trust in physicians		
	Positive	Neutral	Negative
Physician’s personal and practice characteristics			
Country of training	✓ North America/ Western Europe	✓	✓ Eastern Europe
Institution of practice	✓ Reputable	✓	✓ Non-reputable
Years of experience	✓ More	✓	
Being experienced versus up-to-date	✓ Up-to-date	✓	✓
Physician’s gender		✓	
Appearance and hygiene	✓ Professional attire	✓	✓ Unprofessional attire
Physician’s clinical skills			
Being competent	✓ High		✓ Low
Not making mistakes		✓	✓ If major (e.g. led to death)
Educating patients			✓ No education
Interactions with the physician			
Rapport	✓ Good	✓	✓ Bad
Encounter time	✓ Longer time	✓	✓ Shorter time
Financial factors			
Consultation fees		✓	
Free consultation/medication	✓	✓	✓ Suspicion about intention
Being money-oriented	✓ Non-materialistic	✓	✓ Materialistic
Social factors			
Physician being recommended by a family member	✓		
Physician being a family member	✓		
Physician being featured in media	✓	✓	
Physician’s reputation	✓ Good	✓	✓ Bad

### Physician’s personal and practice characteristics

Personal and practice characteristics influencing trust in physicians included: country of training, institution of practice, years of experience, having experience versus being up-to-date, gender, and appearance and hygiene. For most, but not all participants, their trust in the physician depended on that physician’s country of training. For example, many participants have more trust in physicians trained in Western Europe or North America as opposed to Eastern Europe. Similarly, many participants indicated that their trust increased when physicians were working at reputable and private hospitals. One participant explained: “*the doctor should use technologically advanced resources like MRI, and scanners. The hospital where the doctor works is an important factor*” (P_21_).

For some, the physician’s years of experience was a very important determinant of trust, and for a few, it was the most important. However, most participants preferred an up-to-date physician with long years of experience. This was illustrated by a participant saying: “*Even if a physician had several years of experience and he is not following up on the most recent updates, he wouldn’t be on the same level as a fresh graduate who knows much more of the most recent advancements*” (P_22_). Another participant noted the importance of periodically re-evaluating the physician’s competencies as a governmental measure to improve trust. Furthermore, participants reported that the physician’s gender had no influence on their level of trust. On the issue of appearance and hygiene, the majority of participants reported that trust in physicians was influenced by professional attire as one participant explained: “*if I enter a doctor’s clinic and find that it does not reflect a very good hygiene, it would greatly lower my trust in that doctor*” (P_06_).

### Physician’s clinical skills

Physician’s clinical skills mattered. Those included: being competent, not making mistakes, and educating patients. The physician’s competency was one of the most commonly discussed factors influencing the public’s trust in physicians. One participant explained: “*the patient has to trust his/her physician and, therefore, must choose a competent one; you can’t trust someone who’s not competent*” (P_05_). Medical competency was defined in terms of answering inquiries, “*cur[ing] you*” (P_03_) and “*his diagnosis is always correct*” (P_22_). Many participants reported that trust building occurred overtime after several clinical interactions: “*trust is built based on experiences with doctors. You can’t trust them from the beginning; you don’t know them. If I have good results with them, this initiates building trust*” (P_23_). While both the ability of the physician to provide accurate diagnosis and to reach the desire cure were important, many considered the physician’s diagnostic skills as crucial. High competency in diagnosis enhanced trust, while low competency gravely decreased it. Repeated misdiagnosis could drive the patient to stop seeking the physician’s care or to pursue a second opinion.

The majority of participants indicated that minor mistakes did not influence their trust, for example one of the participants said: “*no one is exempt from making mistakes*” (P_16_). However, major mistakes leading to death or disability were certainly a deterrent of trust. For some, the impact of a mistake was tempered whenever the physician admitted committing it and explaining it to the patient.

Many participants indicated that patient education, especially for surgical procedures, was suboptimal, thus leading to decrease in trust: “*if they don’t explain to me what is happening then I would trust them less*” (P_04_). Few participants indicated that physicians should take the initiative to educate their patients, not only regarding their medical condition and treatment outcomes but also regarding any medical or surgical procedures.

### Interactions with the physician

The interactions with the physician theme included factors such as rapport, encounter time, consultation fees, and being money oriented. A lot of the participants emphasized the importance of a good rapport between the physician and the patient; a relationship built on compassion, honesty and respect of patients. The longer the encounter time, the more they trusted their physicians, as this allowed them to discuss concerns and worries. It also allowed the physician to make the right diagnosis, as shown in the voice of this participant: “*when a doctor just looks at a patient and does a quick examination saying nothing is wrong and doesn’t ask or take into account the simple things, she might miss a serious disease. If she gives him a quick look and dismisses him with a prescription, that means she’s not a [good] doctor*” (P_16_).

### Finances

Participants consistently noted that the consultation fees did not affect their trust in or their choice of the physician: “*doctors want to live. They are not “Caritas” [charity]. They help people but for them, this is a job to support their living. They learn to eventually work*” (P_02_).

The majority consented that trust decreased whenever the physician showed signs of being “*materialistic*” (P_24_) also described as “*businessmen*” (P_19_), in contrast to those with the “*more humane*” (P_20_) physicians. However, some participants were in favour of fixed consultation fees, given that some physicians charged exorbitant amounts. Finally, giving free medications by physicians was perceived on a spectrum ranging from positive, to neutral, to negative.

### Social factors

The main social factors mentioned as influencing the individuals’ trust in physicians were: being a family member, recommended by a family member, featured in mainstream media, and/or having a good reputation. All participants said that they trust a physician more who is a family member or who is recommended by a family member. For some, the trust in the physician increased when they made an appearance in the media: “*everyone who goes on the television is good*” (P_12_). For some, a physician’s good reputation increased trust except for one participant who said that: “*some doctors have a good reputation without being good (competent), but some are actually good*” (P_18_).

### Implications

Increased trust in physician led to: [1] being dependent on the physician for referrals; [2] following the physician’s medical advice; [3], continuing to seek the care of the physician despite any medical mistakes, expenses, or being dissatisfied with the institution where the physician practices. Conversely, decreased trust in physicians led to: [1] stopping to seek the physician’s care and [2] seeking a second opinion.

In order to enhance the public’s trust of physicians, participants expected them to: 1- improve patient education: “*I have watched 100s of TV programs where the physician explains to the patient before he treats him, but Lebanese surgeons don’t explain anything*” (P_06_); 2- provide enough encounter time with patients: “*The doctor should provide her patient with enough time and specific things related to each patient’s condition*” (P_09_); and 3- close monitoring by the Ministry of Health. They also suggested that the Order of Physicians impose a fixed consultation fee: “*Theoretically the check-up for all doctors should be fixed*” (P_24_). In addition, they demand that the government periodically reassess the physicians’ competencies.

## Discussion

One of the objectives of this study was to explore the factors associated with trust of members of the public in physicians in the context of the Lebanese healthcare system. To summarize, we found that members of the public trusted experienced physicians who keep abreast with up-to-date evidence, graduated from North America or Western Europe, and worked at a reputable hospital. As for interpersonal skills, being ‘non-materialistic’, having a good rapport with the patient, and providing sufficient encounter time were also important factors associated with trust. Participants showed high levels of trust for physicians featured in the media and those with a good reputation in their social network, as Mechanic and Meyer suggest “trust initially may be based on reputation” [18].

Also, we found that people had higher trust in physicians who are believed to hold the patient’s best interests above everything else. They described the less trustworthy physicians as “*businessmen*” (P_19_) and “*financially oriented*” (P_20_). The physicians’ ethical values were also important traits of trustworthiness. In the Arawi’s study, the physician’s traits most desired by the public were being “honest, humane, ethical, not materialistic, humble/modest, compassionate, respect patient, and God-fearing” [14]. In a recent study in the US, Hwong and colleagues found that physicians who receive high payments from pharmaceutical and medical device industries were perceived as less honest and less committed to the best interest of patients than physicians who received no payments [19]. Moreover, physicians who were perceived by the public as profit-driven, were likely to lose trust among patients. For example, Chinese patients became concerned when a physician gave them a prescription or ordered a medical examination because they did not know if the physician was doing so for the patients’ benefit or for the physician’s own financial gain [20].

We found that clinical competencies, particularly strong diagnostic skills, were an important factor in increasing trust. This was echoed in several studies that reported physician’s competence including thoroughness and knowledge as factors related to trust [18, 21–23]. Given that most patients may have difficulty making an objective assessment of the physician’s technical competence directly [18], some base their opinion on solely the physician’s interpersonal skills [7, 14, 20]. In fact, the physician’s attitude toward patients during their first encounter can contribute to building mutual trust [20]. Thus, even a clinically competent physician but with poor interpersonal skills may likely be perceived as incompetent, thus losing his/her patient’s trust [24]. We also found that patients’ perceptions were affected by the fulfilment of treatment expectations. Thus, a physician with a good therapeutic reputation in the community, especially between relatives, led to a shared perceived trust in that physician’s competence [25].

In Shanghai, China, authors found that the main cause of distrust in physicians was the asymmetry of information between patients and physicians [26]. In another study conducted in Wales and England, shared decision-making and taking the patients’ problems seriously were the two most important factors associated with trust in general practitioners [5]. Furthermore, Thom and Campbell identified “understanding the patient’s individual experience, partnership building, honesty/respect for the patient” as factors determining trust [22]. These results were consistent with our findings that ‘rapport’ and ‘encounter time’ affect trust in physicians.

In the context of the health care system, trust can be classified into two categories: the social trust which relates to the system as a whole, and the interpersonal trust which is a function of a relationship between two parties and the interactions between them [26, 27]. In this paper, we addressed the interpersonal dimension; however, future research is yet to explore the healthcare system as a dimension. Future research should also address the physician’s point of view and potential interventions to enhance trust.

To our knowledge, this is the first study to qualitatively explore the factors which are associated with the trust of the public in physicians in the Middle East region. One strength of this study was the use of quality control approaches such as thorough training of investigators in data collection and data analysis. We also conducted the analysis in a duplicate and independent manner followed by consensus building. Additionally, the sample of participants captured both genders equally, and varied by region and age. One limitation of this study was that participants could not be sampled from all the governorates of Lebanon, but we assume that most findings would likely be shared by other Lebanese. Another limitation is that certain characteristics of participants, such as level of education, may have had an impact on trust and was not captured in this study.

## Conclusion

Trust is one of the central pillars of the physician-patient relationship and has major implications on the patients’ interactions with their physicians. The results of this study revealed five main themes that impacted trust and comprise: the personal and practice characteristics of physicians, clinical skills, interactions with the patient, finances, and several social factors. This is an important step in capturing the unique determinants of trust in Lebanon, especially as the healthcare system continues to change and develop. Reinforcing the physician-patient trust depends on the ability to measure these factors and consequently promote all positive determinants and abolish all threats to the trust relationship. We hope that this study will help promote future research that develops validated tools to measure trust and develop specific interventions and policies to enhance it. Knowing these factors will enable physicians to reflect on their own practice and hopefully help them increase the patients’ trust.

## Additional files

Additional file 1: Questionnaire on the participants’ demographic characteristics entitled “Data collection sheet”. (PDF 36 kb)

Additional file 2: Interview guide that we used to guide the discussion during the semi-structured interviews. It is entitled “Interview guide”. (PDF 39 kb)

Additional file 3: Contains an example of themes, subthemes and codes. (XLSX 14 kb)

## Data Availability

The datasets generated and/or analysed during the current study are not publicly available due to confidentiality of the face-to-face in-depth transcripts but are available from the corresponding author on reasonable request.

## Abbreviations

COREQ: Consolidated Criteria for Reporting Qualitative Research
ID: Identification
MRI: Magnetic resonance imaging
PI: Principal Investigator
US: United States

## References

[1] Möllering G (2001). The nature of trust: from Georg Simmel to a theory of expectation, interpretation and suspension. Sociology..

[2] Simmel G. The philosophy of money: Routledge; 2004.

[3] Meyer S, Ward P, Coveney J, Rogers W (2008). Trust in the health system: an analysis and extension of the social theories of Giddens and Luhmann. Health Sociol Rev.

[4] Pearson SD, Raeke LH (2000). Patients’ trust in physicians: many theories, few measures, and little data. J Gen Intern Med.

[5] Croker JE, Swancutt DR, Roberts MJ, Abel GA, Roland M, Campbell JL (2013). Factors affecting patients’ trust and confidence in GPs: evidence from the English national GP patient survey. BMJ Open.

[6] Skirbekk H, Middelthon AL, Hjortdahl P, Finset A (2011). Mandates of trust in the doctor-patient relationship. Qual Health Res.

[7] Hall MA, Dugan E, Zheng B, Mishra AK (2001). Trust in physicians and medical institutions: what is it, can it be measured, and does it matter?. The milbank quarterly.

[8] Hillen MA, de Haes HC, Smets EM (2011). Cancer patients' trust in their physician—a review. Psycho-Oncology..

[9] Rowe R, Calnan M (2006). Trust relations in health care--the new agenda. Eur J Pub Health.

[10] Huang EC-H, Pu C, Chou Y-J, Huang N (2018). Public Trust in Physicians—Health Care Commodification as a possible deteriorating factor: cross-sectional analysis of 23 countries. INQUIRY: J Health Care Organ, Prov, Financ.

[11] Armstrong K, Rose A, Peters N, Long JA, McMurphy S, Shea JA (2006). Distrust of the health care system and self-reported health in the United States. J Gen Intern Med.

[12] Zheng H (2015). Losing confidence in medicine in an era of medical expansion?. Soc Sci Res.

[13] Zhao D, Zhang Z. Changes in public trust in physicians: empirical evidence from China. Frontiers Med. 2018:1–7. 10.1007/s11684-018-0666-4 30194619

[14] Arawi T (2010). The Lebanese physician: a public's viewpoint. Developing World Bioethics.

[15] Corbin J, Strauss A. Basics of qualitative research: techniques and procedures for developing grounded theory. 3rd ed. Thousand Oaks, CA, US: Sage Publications, Inc; 2008. xv. p. 379–xv.

[16] Tong A, Sainsbury P, Craig J (2007). Consolidated criteria for reporting qualitative research (COREQ): a 32-item checklist for interviews and focus groups. Int J Qual Health Care.

[17] Lincoln YS, Guba YSLEG, Guba EG. Naturalistic Inquiry: SAGE Publications; 1985.

[18] Mechanic D, Meyer S (2000). Concepts of trust among patients with serious illness. Soc Sci Med.

[19] Hwong AR, Sah S, Lehmann LS (2017). The effects of public disclosure of industry payments to physicians on patient trust: a randomized experiment. J Gen Intern Med.

[20] CSc C (2018). Mistrust of physicians in China: society, institution, and interaction as root causes. Developing World Bioethics..

[21] Goold SD, Klipp G (2002). Managed care members talk about trust. Soc Sci Med.

[22] Thom DH, Campbell B (1997). Patient-physician trust: an exploratory study. J Fam Pract.

[23] Thom DH, Hall MA, Pawlson LG (2004). Measuring patients’ trust in physicians when assessing quality of care. Health Aff.

[24] Choy HH, Ismail A (2017). Indicators for medical mistrust in healthcare–a review and standpoint from Southeast Asia. Malaysian J Med Sc: MJMS.

[25] Gopichandran V, Chetlapalli SK (2013). Dimensions and determinants of trust in health care in resource poor settings–a qualitative exploration. PLoS One.

[26] Zhao D-H, Rao K-Q, Zhang Z-R (2016). Patient trust in physicians: empirical evidence from Shanghai, China. Chin Med J.

[27] Liu FR (2013). HMOs and patient Trust in Physicians a Longitudinal Study. Int J Appl Econ.

