# Supplementary material for: Factors associated with the public’s trust in physicians in the context of the Lebanese healthcare system: a qualitative study
Source: BMC Health Serv Res. 2019 Jul 27;19:525. doi: 10.1186/s12913-019-4354-0 (PMC6660947; doi:10.1186/s12913-019-4354-0)
Supplement: Supplementary file 1 — Questionnaire on the participants’ demographic characteristics entitled “Data collection sheet”. (PDF 36 kb) [file 12913_2019_4354_MOESM1_ESM.pdf]

Data collection sheet

Interviewer: ----------------------------- Interview date:----------------------

Recruited from: ☐ Public place (e.g., Super market, Mini-market)

☐ Co-investigator neighborhood

Language of interview: ☐ Arabic ☐ English

1- Sex: ☐ Male ☐ Female

2- Age: ☐ 18-30 ☐ 31-40 ☐ 41-50

☐ 51-60 ☐ >60

3- Level of education: ☐ No education ☐ School ☐ High School

☐ Undergraduate Studies ☐ Masters/PHD

4- Area of residency: ☐ Beirut ☐ North ☐ South

☐ Nabatiye ☐ Bekaa ☐ Mount Lebanon

5- Have been seen by a doctor over the last 4 weeks:

☐ Yes ☐ No
