# Supplementary material for: Factors associated with the public’s trust in physicians in the context of the Lebanese healthcare system: a qualitative study
Source: BMC Health Serv Res. 2019 Jul 27;19:525. doi: 10.1186/s12913-019-4354-0 (PMC6660947; doi:10.1186/s12913-019-4354-0)
Supplement: Supplementary file 2 — Interview guide that we used to guide the discussion during the semi-structured interviews. It is entitled “Interview guide”. (PDF 39 kb) [file 12913_2019_4354_MOESM2_ESM.pdf]

Interview guide

- Can you tell me a little about your experience with doctors?
- Do you feel that people in Lebanon trust physicians in Lebanon?
- Do you personally trust physicians in Lebanon?
- Which factors do you feel positively impact your trust in your doctor?
- Which factors do you feel negatively impact your trust in your doctor?
- What are ways that could improve your trust in your physicians?
